# Multicenter study on ultrasound characteristics of acupoints in the Hand Yangming Meridian and related factors

**DOI:** 10.3389/fmed.2026.1928250

**Published:** 2026-09-23

**Authors:** Fangqin Liu, Yunhao Luo, Jiaojun Yan, Zhengyan Wang, Shiping Wu, Shuting Li, Xuhong Nan, Chunxia Cheng, Xuerong Fu, Yalin Lu, Xuemei Xia, Huan Du, Zhengchao Fan, Lang Qiao

**Affiliations:** 1 School of Medical and Life Sciences, Chengdu University of Traditional Chinese Medicine, Chengdu, China; 2 Department of Ultrasound, Sichuan Integrative Medicine Hospital, Chengdu, China; 3 Department of Ultrasound, West China Hospital of Sichuan University, Chengdu, China; 4 Rehabilitation Medicine Department, Sichuan Integrative Medicine Hospital, Chengdu, China; 5 Department of Ultrasound, Suining Traditional Chinese Medicine Hospital, Suining, Sichuan, China; 6 Department of Ultrasound, Chengdu Integrated TCM & Western Medicine Hospital, Chengdu, China; 7 Department of Ultrasound, Affiliated Hospital of Chengdu University of Traditional Chinese Medicine, Chengdu, China; 8 Department of Ultrasound, The Affiliated Traditional Chinese Medicine Hospital Southwest Medical, Chengdu, China; 9 Department of Ultrasound, The Traditional Chinese Medicine Hospital of Longquanyi, Chengdu, China; 10 Department of Ultrasound, Sichuan Tianfu New Area People's Hospital, Chengdu, China; 11 Department of Ultrasound, Deyang Integrated Traditional Chinese and Western Medicine Hospital, Deyang, China; 12 Department of Ultrasound, Mianyang Hospital of the Traditional Chinese Medicine, Mianyang, China; 13 Department of Ultrasound, Sichuan Second Hospital of The Traditional Chinese Medicine, Chengdu, China

**Keywords:** acupoint, acupuncture, elasticity imaging techniques, mechanism, ultrasonography

## Abstract

**Objective:**

To investigate sonographic characteristics and influencing factors of Hegu (LI 4), Xialian (LI 8) and Shousanli (LI 10) on the Hand-Yangming Large Intestine Meridian via multimodal ultrasonography.

**Methods:**

In total of 110 healthy volunteers were recruited across multiple centers, with standardized localization of Hegu(HG, LI 4), Xialian(XL, LI 8) andshousanli(SSL, LI 10) performed. Grayscale ultrasonography, color Doppler flow imaging and shear wave elastography were used for ultrasonic data acquisition. Needle tip depth, acupoint-bone adjacent distance, tissue composition, blood perfusion and biomechanical properties at acupoints were quantitatively detected.

**Results:**

Baseline information and observational indices were consistent among research centers (*P* > 0.05). No correlations were identified between acupoint ultrasonic parameters and volunteers' baseline characteristics (*P* > 0.05). The median skin depth and mid-radius distance were 1.72 cm (1.38, 1.96 cm) and 0.93 cm (0.63, 1.24 cm) for LI 4; 1.64 cm (1.36, 1.95 cm) and 0.43 cm (0.29, 0.60 cm) for LI 8 1.75 cm (1.51, 2.14 cm) and 0.66 cm (0.45, 0.84 cm) for LI 10. Anatomically, LI 4 was mainly located in adductor pollicis muscle (62.7%), LI 8 in supinator muscle (41.8%), and LI 10 in the fascia between extensor carpi radialis longus and supinator muscles (39.1%). All three acupoints were closely associated with fascia structures (*χ²* = 11.864, *P* = 0.003)., and presented higher elastic modulus than surrounding reference tissues (*P* < 0.05).

**Conclusions:**

Acupoints of the Hand-Yangming Large Intestine Meridian have specific ultrasonic manifestations, including definite anatomical location, tight fascial attachment, increasing fascial correlation along the meridian, and superior local tissue biomechanics. This study provides quantitative ultrasonic evidence for the meridian-connective tissue hypothesis, and offers a practical method for exploring acupuncture mechanisms.

## 1 Introduction

Meridian theory has guided clinical practice for millennia and remains pivotal in diagnosis and treatment. However, its theoretical framework differs fundamentally from that of modern medicine, making the identification of connections between the two a primary research focus (1). Ultrasound has been increasingly applied to acupuncture research, yet the current knowledge of acupoint ultrasonography remains largely descriptive. Most studies employ ultrasound primarily for needle guidance, safety assurance, or gross efficacy assessment, whereas systematic investigations into the intrinsic features of acupoints—such as tissue architecture and muscle composition, microvascular flow, and tissue elasticity—are scarce. Standardized imaging protocols and validated quantitative metrics are lacking, severely undermining cross-study comparability and clinical translatability. To address these gaps, the present multicenter, large-sample study systematically characterizes the ultrasonographic features of acupoints across diverse body habitus, establishes standardized acquisition and measurement procedures, and explores the mechanistic basis of acupoint-specific ultrasound signatures, thereby advancing the field from descriptive observation toward mechanism-informed, clinically applicable knowledge. The meridian-connective tissue correlation hypothesis is a prominent theory, with substantial evidence demonstrating a close association between meridians and fascial tissues. Acupoints represent critical nodes in traditional meridian theory and have emerged as an active focus of modern medical investigation (2, 3). In this study, three specific acupoints on the Hand-Yangming Large Intestine Meridian—LI 4, LI 8, and LI 10—were selected based on several considerations. First, our team has maintained a long-standing research focus on this meridian, accumulating considerable experience through the development of an ultrasound-based method for acupoint observation, the discovery of potential relationships between acupoints and anatomical structures, and the identification of promising biomechanical research prospects, all of which reflect a desire to progressively deepen our understanding of its properties. Second, LI 4 (Hegu, HG) is a principal and highly representative acupoint on this meridian, playing a pivotal role in clinical acupuncture. LI 8 (Xialian, XL) and LI 10 (Shousanli, SSL) were chosen because they are adjacent to yet at a discernible distance from LI 4, offering an opportunity to explore the regularities and characteristics of acupoints along the Hand-Yangming meridian. Third, the forearm region allows convenient ultrasonographic observation and offers a high degree of needling safety, thereby enhancing participant compliance—an important practical consideration underlying our selection. Acupoint research is inherently multidisciplinary, with imaging studies constituting a vital component (4, 5). Recently, ultrasonography has emerged as a novel investigative technology in this field. High-frequency ultrasound provides real-time, dynamic visualization of fine structures, and its multimodal capabilities offer substantial potential for acupoint research (6–8). In this exploratory study, we selected three acupoints on the right Large Intestine Meridian of Hand-Yangming—Hegu (LI 4), Xialian (LI 8), and Shousanli (LI 10) (9, 10), A multicenter large-sample observation was performed via ultrasonic imaging to characterize the local ultrasonic manifestations of targeted acupoints, clarify their corresponding anatomical structures and investigate the localized action mechanisms of meridian-acupoint system. This work is expected to provide novel evidence and technical approaches for imaging-based modern research of meridians and acupoints.

## 2 Methods

### 2.1 Study design

This prospective, multicenter study was approved by the Ethics Committee of Sichuan Provincial Hospital of Integrated Traditional Chinese and Western Medicine (Approval No. KY-2023-010). A total of 110 healthy volunteers were publicly recruited from July 2024 to July 2025 across 6 medical centers. A total of 10 clinical centers were initially planned to participate in this study. Due to the real-world clinical constraints, valid data were ultimately obtained from 6 centers, with participant enrollment numbers stratified by study center summarized (Supplementary Table S1). All enrolled volunteers received standardized acupuncture at three acupoints of the Large Intestine Meridian of Hand-Yangming, including Hegu (LI 4), Xialian (LI 8), and Shousanli (LI 10), and underwent standardized multimodal ultrasound examination simultaneously. All participants provided written informed consent prior to enrollment, and their demographic information and medical history were collected systematically (9).

### 2.2 Intervention protocol and operating specifications

Each participating center was equipped with an ultrasonic diagnostic system supporting high-frequency linear array probes and shear wave elastography (SWE): Mindray Resona 9 with L15-3WU probe or Mindray Resona 7 with L14-3WU probe. Acupuncture needles (0.25 mm × 40 mm) were supplied by Suzhou Medical Supplies Factory Company Limited, All primary operators at each center were full-time physicians with more than 5 years of clinical experience in both acupuncture manipulation and ultrasonic diagnosis. Uniform standardized training was conducted for all operators prior to study initiation, and ongoing operational supervision was implemented throughout the research period.

Acupoint localization and marking were performed in accordance with the WHO Standard Acupuncture Point Locations, and a perpendicular insertion technique was uniformly adopted for all acupoints (11–14). To facilitate manipulation and measurement and guarantee the feasibility and safety of this exploratory trial, only acupoints on the right forearm were selected for all subjects, namely Hegu (HG, LI 4), Xialian (XL, LI 8) and Shousanli (SSL, LI 10). LI 4 is located on the dorsum of the hand, at the midpoint of the radial side of the second metacarpal bone. LI 10 is located on the posterolateral aspect of the forearm, on the line connecting LI 15 (Jianyu) and LI 11 (Quchi), 2 B-cun distal to the transverse cubital crease. LI 8 is located on the radial side of the dorsal forearm, at the junction of the posterior one-third of the line connecting LI 5 (Yangxi) and LI 11 (Quchi) (Figure 1). Deqi was defined as subjects' subjective soreness, numbness and distending sensation or distinct dragging resistance felt by acupuncturists during needling (2). Ultrasonic images were acquired using an out-of-plane approach with a transverse scanning plane relative to the acupoint. On these images, the acupoint region was defined as a cubic volume of 1 mm × 1 mm × 1 mm centered at the needle tip upon deqi achievement; the reference site was set at a position 5 mm horizontally left to the targeted acupoint (15).

**Figure 1:**
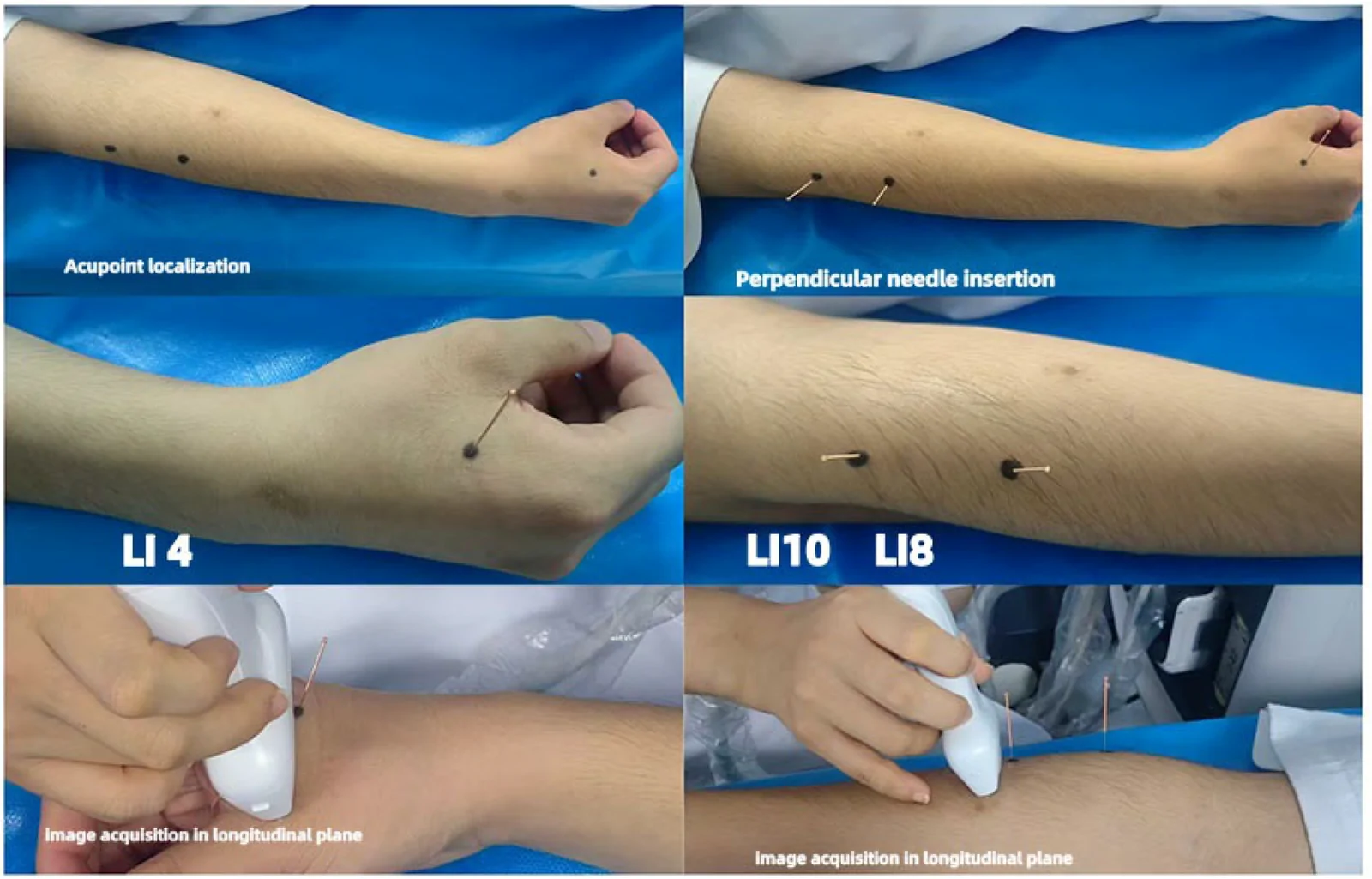
Diagram of acupoint manipulation.

To ensure consistency of acupoint localization across the ten centers, a standardized training and qualification procedure was implemented before formal data collection. All operators underwent uniform, hands-on training in acupoint identification and ultrasound probe positioning. Each operator was required to complete at least 20 practice localizations and was permitted to participate in the study only after their technique was evaluated and found to comply fully with the study protocol. Pre-study training sessions were documented photographically (Supplementary Figure S1)

### 2.3 Inclusion and exclusion criteria

**Inclusion criteria**: Healthy adult subjects aged 18–70 years (16), with intact somatosensory function, clear consciousness and unimpeded verbal communication.

**Exclusion criteria**: Subjects with coagulation disorders, sensory or speech dysfunction, or a history of acupuncture syncope; marked hypersensitivity or severe phobia to acupuncture; previous trauma, surgery or anatomical abnormalities on the right forearm; incomplete acupoint ultrasonic images failing quality assessment; missing basic demographic data; pregnant or lactating females; receipt of acupuncture or physical therapy on the right forearm within the preceding week.

### 2.4 Operating procedures and data collection

Qualified acupuncturists performed needling at the three predefined acupoints until Deqi was elicited, while sonographers captured ultrasonic images (Figure 2) and marked the needle tip as the acupoint location (Figure 1). At Hegu (HG, LI 4): the vertical depth from acupoint to skin (Vertical) and the perpendicular horizontal distance from the acupoint to the midpoint on the radial side of the second metacarpal bone (Level) were measured separately. At Xialian (XL, LI 8): the vertical depth from acupoint to skin (Vertical) and the perpendicular distance to the radial bone plane (Level) were measured separately. At Shousanli (SSL, LI 10): the vertical depth from acupoint to skin (Vertical) and the perpendicular distance to the radial bone plane (Level) were measured separately (17). Color Doppler flow imaging was conducted at each acupoint to assess blood perfusion. Immediately after needle removal, shear wave elastography was applied to acquire Young's modulus values at both acupoint and reference sites (15). All final measured parameters were calculated as the average of three repeated measurements.

**Figure 2:**
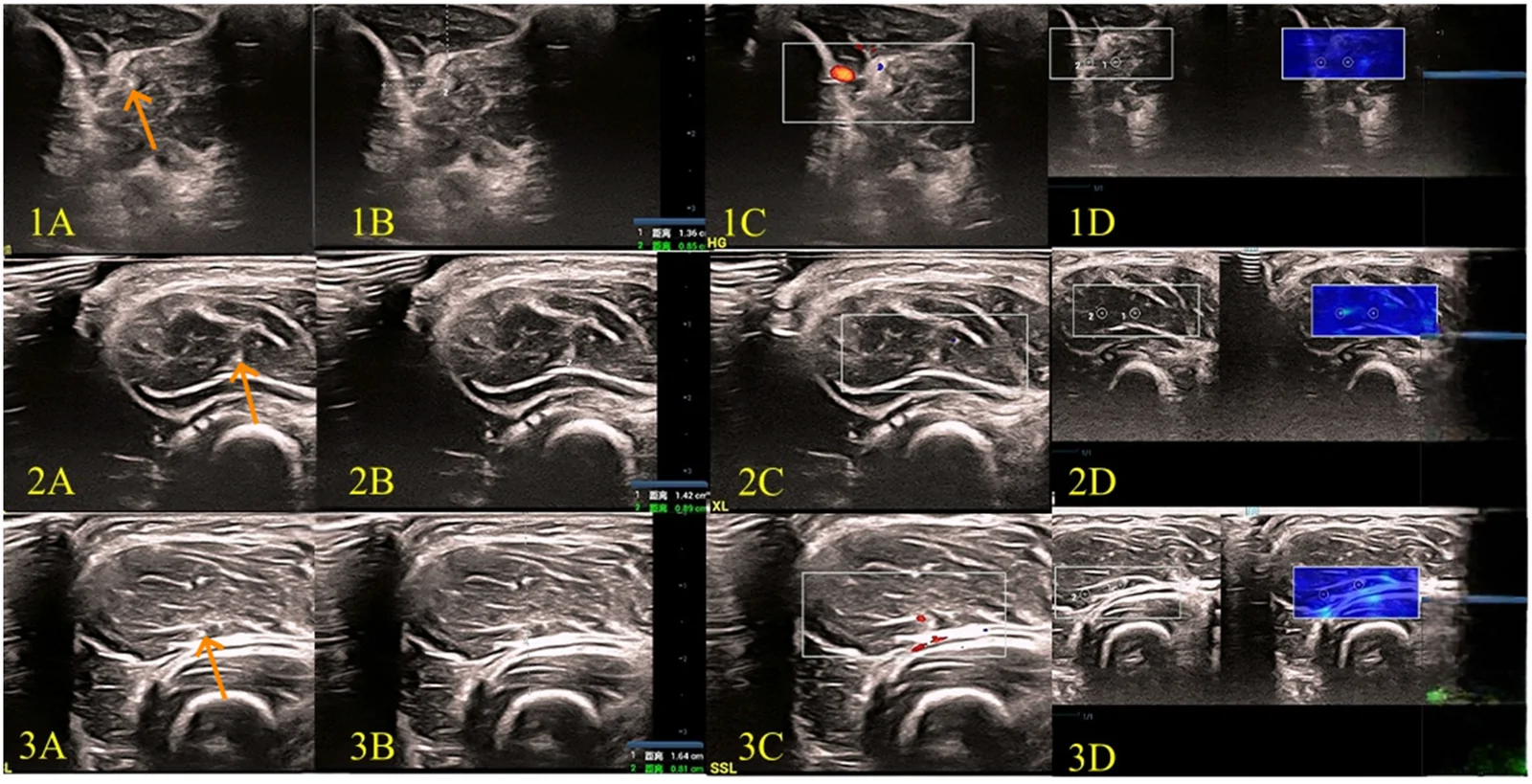
Ultrasonic image acquisition of acupoints. Arrows indicate the locations of target acupoints: **(1)**: Hegu (LI 4), **(2)**: Xialian (LI 8), **(3)**: Shousanli (LI 10), A: grayscale ultrasound, B: dimensional measurement image of acupoint, C: CDFI, D: SWE.

### 2.5 Image and data evaluation

Two independent researchers sorted raw data and excluded subjects with incomplete records or poor-quality ultrasonic images. Two experienced physicians performed blind image review to reach consensus for acupoint localization and blood flow findings; inconsistent interpretations were resolved via group discussion, and unresolved disputes were adjudicated by the corresponding author ultimately.

All enrolled acupoints (Hegu, Xialian, Shousanli) were divided into three respective groups. Anatomical structures identifiable via two-dimensional ultrasound at acupoints mainly comprised muscles and fasciae. Muscles were stratified into superficial and deep layers along the vertical direction from the cutaneous surface. For Hegu acupoint: the superficial muscle was the first dorsal interosseous muscle and the deep muscle was adductor pollicis. Xialian and Shousanli shared similar anatomical constitution, with extensor carpi radialis longus as the superficial muscle and supinator as the deep muscle. When the acupoint was situated inside a muscle, the perpendicular plumb line from skin crossing the acupoint within the target muscle was equally trisected to define superficial, middle and deep muscular portions. Acupoints were categorized as fascia-associated or non-fascia-associated, and further distinguished between epimysium and perimysium. CDFI was graded according to the Adler blood flow classification standard. Mean Young's modulus values of acupoint and reference sites (defined as a fixed 5 mm horizontal offset from each acupoint) were collected under five-star image quality control criteria.

### 2.6 Statistical analysis

Statistical analyses were conducted using SPSS 25(IBM, USA).The Shapiro–Wilk test was used to assess normality of continuous variables. Non-normally distributed measurement data were expressed as median (interquartile range) [M (Q1, Q3)] and compared using the Kruskal–Wallis H test. Categorical variables were presented as frequency (percentage) and compared by the chi-square test or Fisher's exact test, as appropriate. Pairwise comparisons of proportions among the three groups were conducted using the partition of chi-square test with Bonferroni correction. A two-sided *P* < 0.05 was considered statistically significant.

## 3 Results

### 3.1 Baseline characteristics of subjects

The research flowchart is presented (Figure 3). Consistency verification was implemented for data from all participating sub-centers. Baseline indicators including age, gender composition, height and body weight were well balanced among subjects across different centers (Table 1), with no statistically significant inter-group differences *P* > 0.05. No significant between-center differences were found in observational parameters such as acupoint dimensional measurements, blood flow grading and mean Young's modulus values *P* > 0.05.

**Figure 3:**
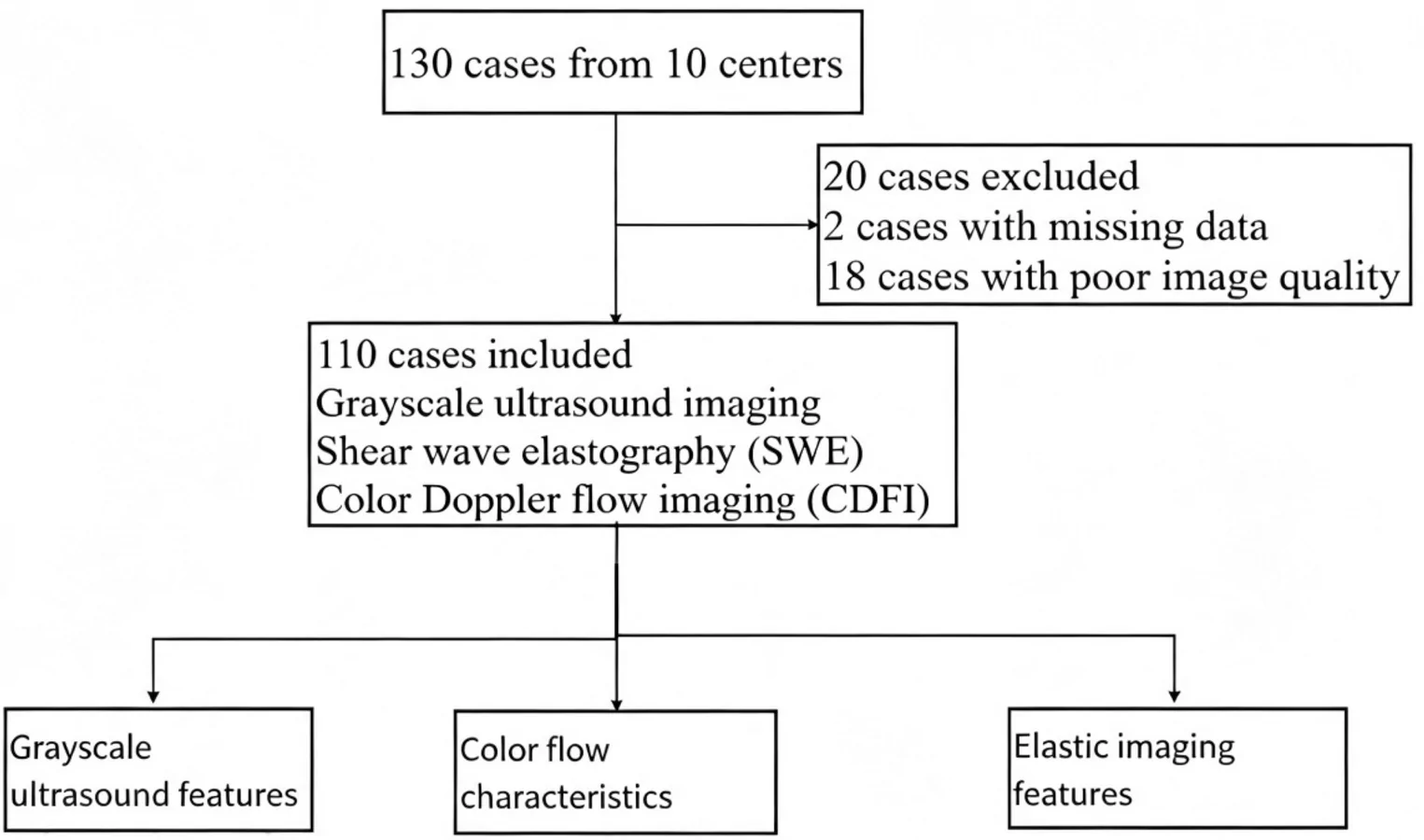
Flowchart of the study.

**Table 1:** Baseline demographic and clinical characteristics of the study population.

Variable	Total (*N* = 110)
Age (years), Median (Q_1_, Q_3_)	25 (21, 31)
Height (cm), Median (Q_1_, Q_3_)	164 (158, 168)
Weight (kg), Median (Q_1_, Q_3_)	58 (51, 63)
Body mass index (BMI) (kg/m²), Median (Q_1_, Q_3_)	21.63 (18.67, 23.71)
Sex, *n* (%)	
Female	68 (61.8)
Male	42 (38.2)

Correlation analyses between observational indexes (dimensional parameters, acupoint location, blood flow status and Young's modulus) and demographic data (gender, age, height, body weight) of the three acupoint groups revealed the following results: for Hegu (LI 4), gender and height were significantly correlated with partial measurement indicators and average Young's modulus *P* < 0.05; for Xialian (LI 8), body weight was significantly associated with local blood flow *P* < 0.05; no statistically significant correlations existed between all detected parameters and general demographic data for Shousanli (LI 10) *P* > 0.05. All statistically significant correlation coefficients had absolute values below 0.4, indicating weak correlations.

### 3.2 Two-dimensional ultrasonography

#### 3.2.1 Acupoint measurement parameters

Two distance indicators were measured: the vertical depth from acupoint to skin (Vertical), and the perpendicular distance from acupoint to the midpoint of the radius (Level). Scatter plots revealed periodically fluctuating and gradually converging distribution patterns of these measurements across the three acupoint groups, median values [interquartile range] were listed as follows: Hegu (HG): Vertical = 1.72 (1.38, 1.96) cm, Level = 0.93 (0.63, 1.24) cm, Xialian (XL): Vertical = 1.64 (1.36, 1.95) cm, Level = 0.43 (0.29, 0.60) cm，Shousanli (SSL): Vertical = 1.75 (1.51, 2.14) cm, Level = 0.66 (0.45, 0.84) cm.

#### 3.2.2 Anatomical location of target acupoints

Along the course of the Large Intestine Meridian of Hand-Yangming, significant anatomical discrepancies were detected between Hegu and the other two adjacent acupoints (Xialian and Shousanli) *P* < 0.05. No statistically significant difference in anatomical composition was found between Xialian and Shousanli, which are anatomically close to each other *P* > 0.05. Hegu was predominantly located within the adductor pollicis muscle (62.7%), among which 29.1% lay in the superficial layer of adductor pollicis (Table 2). Xialian mostly resided in the supinator muscle (41.8%), with 20.9% positioned in the superficial supinator. Shousanli was primarily situated at intermuscular fascia (39.1%), and 37.3% of this acupoint was found within the fascia between extensor carpi radialis longus and supinator (Figure 4). These findings reveal a distinct distribution pattern of connective tissue, particularly intermuscular fascia, along the course of the Large Intestine Meridian, with a proximal increasing involvement from Hegu to Shousanli; this heterogeneity provides morphological evidence supporting the meridian-connective tissue hypothesis elaborated in the Discussion.

**Table 2:** Analysis of anatomical structures at acupoint locations.

Variables	Hegu*^,^**	Xialian*^,^***	Shousanli**^,^***	Statistic	*P*
Dissect, *n* (%)				$\chi^{2}=25.5$	＜0.001
Superficial muscle layer	14 (12.7)	29 (26.4)	34 (30.9)		
Deep muscle laye	69 (62.7)	46 (41.8)	33 (30.0)		
connective tissue	27 (24.5)	35 (33.3)	43 (39.1)		

* *P* = 0.004.

** *P* = 0.001.

*** *P* = 0.187.

**Figure 4:**
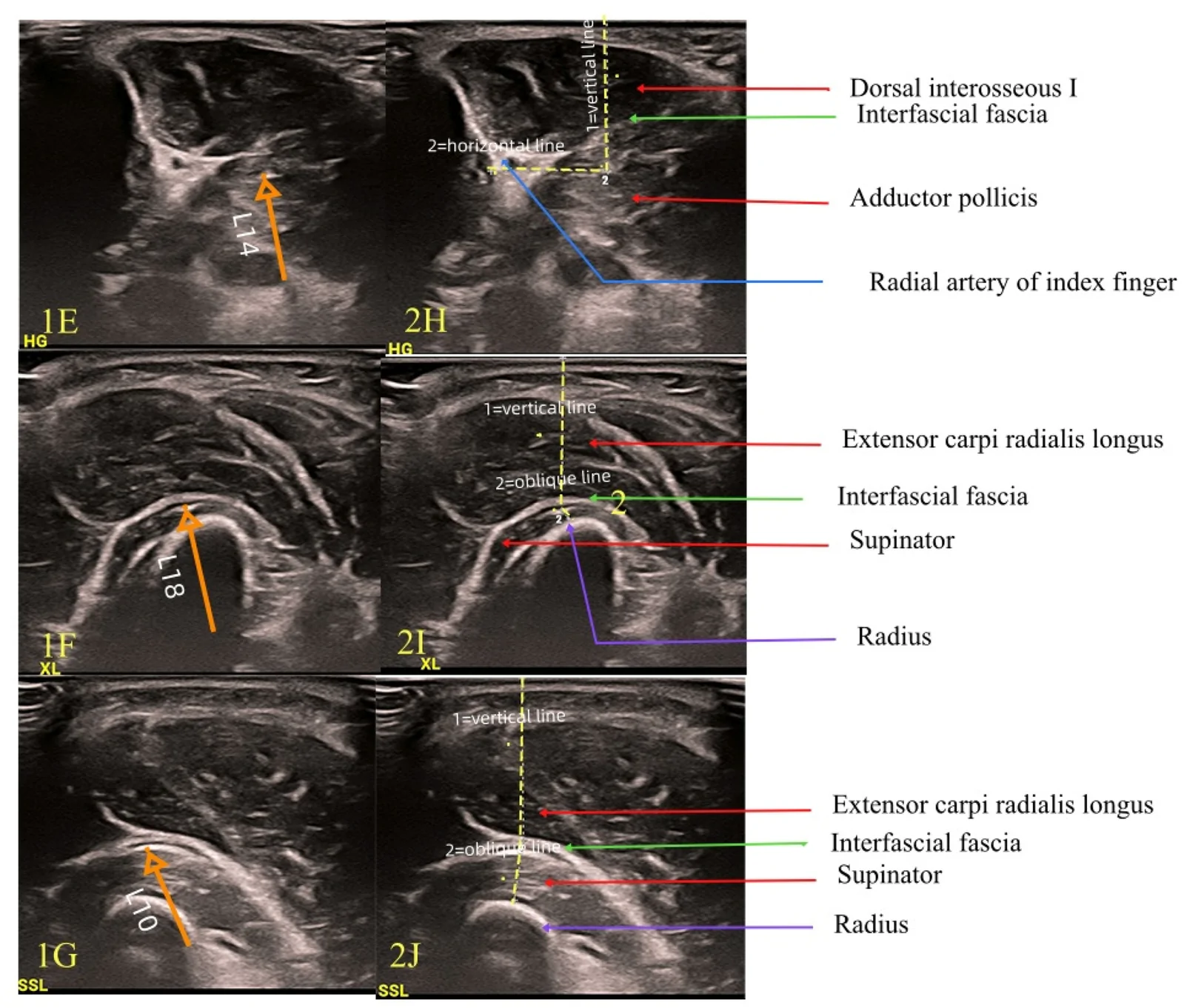
Transverse ultrasound scans: acupoint location and anatomical distribution. yellow dashed lines **(1)** and **(2)**: measurement lines starting from acupoint locations. Arrows indicate the locations of target acupoints: 1E: Hegu (LI 4), 1F: Xialian (LI 8), 1G: Shousanli (LI 10). **(1)**: Grayscale ultrasound image of acupoint; **(2)**: Acupoint measurement image.

#### 3.2.3 Correlation between acupoint location and fascia

Obvious deqi sensation could be induced when the needle tip touched fascia, indicating a close association between acupoint positioning and fascial tissues. The proportion of acupoints situated on fascia differed significantly among the three groups (*P* = 0.003). The rates of fascia-associated acupoints were 50.9% for Hegu, 68.2% for Xialian, and 71.8% for Shousanli (Table 3).

**Table 3:** Analysis of the correlation between acupoints and fascia.

Comparison of acupoint parameter (fascial/non-fascia)				
Variables	Fascial (*n* = 208 )	Non-fascia (*n* = 122 )	Statistic	*P*
Local			11.864	0.003
Hegu^+*^	56 (50.9)	54 (49.1)		
Xialian^#*^	75 (68.2)	35 (31.8)		
Shousanli^#+^	79 (71.8)	31 (28.2)		

+Pairwise comparison between HG and SSL, *P* = 0.03;

*Pairwise comparison between HG and XL, *P* = 0.03;

#Pairwise comparison between XL and SSL, *P*＞0.05.

### 3.3 Shear wave elastography

Significant differences in mean elastic modulus between acupoint sites and reference sites were observed at Shousanli acupoint (*P* < 0.05). The corresponding P values of Hegu and Xialian were (*P* = 0.048) and (*P* = 0.047), respectively, suggesting statistically significant differences in elastic modulus between acupoint and reference positions for Hegu and Xialian. In general, the median value of elastic modulus was higher at acupoint sites than at reference sites (Table 4).

**Table 4:** Comparison of mean elastic modulus between acupoint site and reference site.

Variables	Acupoint site	Reference site	Statistic	*P*
Hegu	2.76 (2.20, 3.29)	2.66 (2.06, 3.30)	6.038	0.048
Xialian	2.86 (2.25, 3.49)	2.79 (2.17, 3.32)	6.030	0.047
Shousanli	2.83 (2.36, 3.36)	2.69 (2.19, 3.16)	6.797	0.033

### 3.4 Color Doppler flow imaging

According to Adler grading of blood flow signals on color Doppler ultrasonography, most acupoint regions presented no detectable blood flow. Pairwise comparisons of blood flow grading among the three acupoints showed no statistically significant differences *P* > 0.05 (Table 5).

**Table 5:** Blood flow grading of acupoints by color Doppler ultrasonography.

Blood flow	No obvious	Sparse punctate	Moderate strip-shaped	Statistic	*P*
Acupoint, *n*(%)				–	0.438
Hegu	97 (88.2)	10 (9.1)	3 (2.7)		
Xialian	103 (92.7)	7 (6.4)	1 (0.9)		
Shousanli	98 (89.1)	11 (10.0)	1 (0.9)		

–, Fisher's exact test.

## 4 Discussion

In the present study, to most faithfully reflect and investigate traditional meridian theory, we adopted the fundamental principle of clinical acupuncture practice—using deqi as the criterion for acupoint identification, encompassing both the subject's sensations of soreness, numbness, and distension and the practitioner's perception of needle grasping. Multimodal ultrasonography was applied to observe Hegu, Xialian and Shousanli acupoints of the Hand-Yangming Large Intestine Meridian. Ultrasonic features including anatomical location, tissue composition and biomechanical properties of the three acupoints were systematically analyzed. This study partially elucidates the correlations between acupoints on the Hand-Yangming Large Intestine Meridian of the right hand and forearm and their anatomical structures, as well as the biomechanical responses induced by acupuncture. The findings provide experimental evidence for further researches on meridian characteristics and the underlying mechanism of acupoints.

### 4.1 Baseline demographics and ultrasonic characteristics of acupoints

No definite correlation was identified between subject baseline characteristics and acupoint ultrasonic parameters. Correlation analyses between demographic data and measured indicators of three acupoints (Hegu, Xialian, Shousanli) revealed statistically significant correlations at *P* < 0.05 in several items: gender and height were correlated with partial location parameters and mean Young's modulus for Hegu; body weight was correlated with blood flow grading for Xialian. Nevertheless, all absolute correlation coefficients of these statistically significant associations were less than 0.4, indicating weak correlations.

These results indicated that despite statistical significance for partial indices, the practical association strength remained very low, and baseline demographics (gender, age, height, body weight) contributed minimally to the variation of acupoint measurements. Accordingly, ultrasonic parameters of the three acupoints were generally independent of subject baseline features, with negligible practical influences from demographic factors. Demographic covariates do not need to be treated as major confounding factors for adjustment or stratified analysis in subsequent mechanistic exploration and clinical research.

### 4.2 Grayscale ultrasonography

The measurement data of acupoint sites demonstrated a relatively concentrated spatial distribution for each acupoint. Although subtle interferences from confounding factors may exist, the measured parameters of the three acupoints presented stable distribution patterns, with most values concentrated around the median level. This finding is inconsistent with the traditional view that acupuncture efficacy derives from individual subjective sensations (18). These findings support the hypothesis that the functional specificity of acupoints may originate from their unique anatomical structural basis.

Further subgroup analysis confirmed the preferential localization of the three acupoints in distinct tissue-specific structures. Hegu was predominantly embedded in the adductor pollicis muscle, Xialian primarily resided within the supinator muscle, and Shousanli was mostly positioned in the intermuscular fascia. This targeted distribution pattern closely matches the local anatomical microenvironment of each acupoint, further indicating that the functional specificity of acupoints is essentially derived from their unique anatomical structural foundations (19).

Among anatomical structures visualised on high-frequency ultrasound, acupoints show robust anatomical connections to fascia, namely intermuscular fascia and perimysium. Experimental observations consistently demonstrated that needle contact with fascial tissues substantially increases the probability of deqi induction in participants (20). Notably, fascia cannot be fully visualized on ultrasonic scans; acupoints categorized into the non-fascia group may still overlay microscopic fascial fibres. Comparative quantification of fascial occupancy across the three acupoints revealed a progressive increase in fascial proportion following the proximal meridian course. Such anatomical trends align with classical meridian doctrines and corroborate the fascial theory postulating topographical correspondence between meridian pathways and systemic fascial networks (19–21). As an interconnected three-dimensional network of connective tissue, fascia not only provides mechanical structural support for acupoints but may also mediate local signal transduction and physiological regulation via embedded nerve terminals, capillary vasculature and resident immune cells (22–25). Thereby, fascia plays a pivotal role in the generation of acupuncture deqi sensations and therapeutic efficacy. Accordingly, this study suggests that fascial structures constitute a core but not exclusive anatomical component of acupoint-meridian systems. The correlation between meridian trajectories and fascial distribution varies across different segments, with a gradual increase in fascial overlap along the ascending course of the meridian. These findings offer novel insights for ultrasonographic and meridian research. The identified progressive anatomical pattern of fascia-associated distribution among forearm acupoints of the Hand-Yangming Meridian provides critical morphological evidence and a structural carrier for the energy transmission and neuromuscular reflex characteristics of propagated meridian sensation, which have been validated in previous studies (26–28).

Ultrasound enables clear and accurate localization of deqi sites and identification of the dominant innervating nerves in corresponding regions. In the present study, the deqi site at Hegu is primarily innervated by the deep branch of the ulnar nerve, while xialian and Shousanli are supplied by the radial nerve. This innervation pattern is consistent with previous anatomical findings, indicating that distinct innervation distributions among acupoints constitute an essential anatomical basis for the neuromodulatory mechanism underlying acupuncture efficacy (29, 30). These findings provide novel imaging evidence for individualized research on the neuromodulatory mechanism of clinical acupuncture and may facilitate further investigations into the neural regulatory mechanism of acupuncture therapy

### 4.3 Color Doppler flow imaging

All acupoints are located within superficial soft tissues. On high-frequency ultrasonography with color Doppler, most acupoint regions present hypovascularity with blood flow grades ranging from 0 to 1. Previous studies have demonstrated that acupuncture intervention can induce alterations in local microcirculation (31–33). However, the present study revealed no remarkable differences in color blood flow among distinct acupoints or between acupoints and control points, which may be attributed to the sensitivity limitation of the adopted blood flow detection technique. Apart from color Doppler, other ultrasonic modalities for regional blood flow evaluation include superb microvascular imaging and contrast-enhanced ultrasonography (CEUS). In particular, contrast-enhanced ultrasonography exhibits prominent superiority in assessing local circulation. Nevertheless, all subjects enrolled in this trial were healthy volunteers, and the application of contrast-enhanced ultrasonography was restricted due to ethical considerations (34, 35). Our team is conducting additional relevant experiments for further exploration.

### 4.4 Shear wave elastography

In this study, shear wave elastography was used to quantitatively compare the mean Young's modulus values between acupoints and control sites. The results showed that the average elastic modulus of the three acupoints was significantly higher than that of the control points. Numerous previous studies have suggested that acupuncture stimulation at acupoints may trigger discharges of tiny regional nerves and subsequent contraction of soft tissues, leading to an elevation in Young's modulus. Accordingly, elastographic features manifest as increased stiffness at the central acupoint area with a gradual decline in stiffness outward from the center (36). Meanwhile, such discrepancies may also result from denser fascial tissues within acupoint regions (37), as well as denser nerve terminals and microvascular networks (20, 38), and inherent specificity in the mechanical properties of local tissues (36). Shear wave elastography provides a potential quantitative imaging biomarker for the objective identification of acupoints (39), further verifying the distinguishable tissue characteristics of acupoints.

## 5 Limitations

This study is a preliminary multicenter exploration of *in vivo* acupoint observation using multimodal ultrasonography. Further optimization is still required for color Doppler detection techniques. Acupoint variations under different physiological/pathological conditions were not investigated, and no patient cohorts were included as control groups，Additionally, only the right forearm was examined, which precludes assessment of bilateral consistency of the observed acupoint characteristics. These limitations restricts the extrapolation of research findings and points out directions for follow-up research.

## 6 Conclusions

Hegu (LI 4), Xialian (LI 8) and Shousanli (LI 10) on the Large Intestine Meridian of Hand-Yangming present distinctive ultrasonic features, with their locations concentrated within specific muscular or fascial structures. Along the proximal course of the meridian, the correlation with fascia increases progressively. From a biomechanical perspective, acupoints possess unique tissue properties with higher stiffness compared with control sites. The visualized and quantitative ultrasonic assessment protocol established herein supplies imaging and biomechanical evidence for the meridian-connective tissue hypothesis, and lays a novel. methodological foundation for exploring acupuncture mechanisms and the integration of traditional Chinese and Western medicine.

## Data Availability

The original contributions presented in the study are included in the article/**Supplementary Material**, further inquiries can be directed to the corresponding author.
