# Supplementary material for: Multicenter study on ultrasound characteristics of acupoints in the Hand Yangming Meridian and related factors
Source: Front Med (Lausanne). 2026 Sep 23;13:1928250. doi: 10.3389/fmed.2026.1928250 (PMC13642146; doi:10.3389/fmed.2026.1928250)
Supplement: Supplementary file 1 [file Supplementaryfile1.pdf]

Supplementary Table:

Supplementary Table 1. Summary of Enrollment by Center.

ID	number (n)
1	28
2	18
3	8
4	12
5	16
10	28
6	0
7	0
8	0
9	0

Supplementary Table 2. Correlations between observation indicators of Hegu (LI 4) acupoint and general characteristics.

HG-Correlations										
		**HGver<br>tical**	**HGlevel**	**needlet<br>ipT**	**detailed<br>L**	**needlet<br>ipP**	**detaile<br>dM**	**CDFI**	**MEAN<br>11**	**MEA<br>N12**
year	*r*	-0.072	0.055	0.041	0.046	0.165	-0.060	-0.105	0.126	0.131
	P	0.457	0.570	0.671	0.631	0.084	0.534	0.274	0.189	0.171
high	*r*	0.218	0.023	-0.323	-0.296	0.112	-0.166	0.165	0.098	-0.037
	P	0.128	0.870	0.021*	0.035*	0.434	0.243	0.247	0.492	0.795
weigh	*r*	0.187	-0.055	-0.210	-0.188	-0.054	-0.124	0.095	0.306	0.128
t	*P*	0.193	0.702	0.138	0.186	0.705	0.385	0.509	0.029*	0.370
sex	*r*	0.289	0.189	-0.111	-0.049	0.133	0.020	0.110	0.044	0.005
	P	0.002	0.047*	0.246	0.611	0.163	0.837	0.249	0.648	0.960

Data are presented as correlation coefficient r and P value. * $P < 0.05$ indicates a significant correlation. vertical: vertical distance from the acupoint to the skin; +: vertical distance from the acupoint to the radius; needletipL, detailedL, needletipR, and detailedM: acupoint locations; CDFI: blood flow condition; MEAN: mean elasticity value; 1: acupoint site, 2: reference site.

Supplementary Table 3. Correlations between observation indicators of Xialian (LI 8) acupoint and general characteristics.

XL-Correlations

		XLvertical	XLlevel	needletipT	detailedL	needletipP	detailedM	CDFI	MEAN11	MEAN12
year	*r*	-0.032	0.161	-0.021	-0.006	-0.073	-0.007	0.181	0.078	0.131
	P	0.741	0.092	0.832	0.949	0.449	0.945	0.057	0.414	0.171
high	*r*	0.121	-0.061	-0.036	-0.044	0.088	-0.014	0.230	-0.037	-0.037
	P	0.399	0.669	0.803	0.762	0.544	0.924	0.105	0.799	0.795
weight	*r*	0.198	0.103	-0.128	-0.108	0.066	-0.081	0.394	0.056	0.128
	P	0.163	0.471	0.375	0.456	0.651	0.577	0.004*	0.696	0.370
sex	*r*	0.167	0.001	0.066	0.115	-0.036	0.140	0.036	0.000	0.005
	P	0.081	0.992	0.495	0.232	0.712	0.143	0.705	0.999	0.960

Data are presented as correlation coefficient r and P value. * *P* < 0.05 indicates a significant correlation. vertical: vertical distance from the acupoint to the skin; +: vertical distance from the acupoint to the radius; needletipL, detailedL, needletipR, and detailedM: acupoint locations; CDFI: blood flow condition; MEAN: mean elasticity value; 1: acupoint site, 2: reference site.

Supplementary Table 4. Correlations between observation indicators of Xialian (LI 8) acupoint and
general characteristics.

SSL-Correlations

		SSLvertical	SSLlevel	needletipT	detailedL	needletipP	detailedM	CDFI	MEAN11	MEAN12
year	*r*	-0.022	-0.005	-0.040	-0.056	0.087	-0.048	0.043	0.047	0.075
	P	0.822	0.961	0.675	0.561	0.369	0.616	0.655	0.624	0.436
high	*r*	0.216	0.092	-0.022	-0.043	-0.005	-0.046	-0.037	0.083	-0.025
	P	0.132	0.525	0.881	0.767	0.972	0.746	0.796	0.562	0.861
weight	*r*	0.235	0.123	-0.049	-0.209	-0.135	-0.218	0.104	0.080	0.086
	P	0.100	0.394	0.732	0.140	0.346	0.124	0.466	0.577	0.548
sex	*r*	0.097	0.052	0.061	0.019	-0.064	0.035	-0.102	-0.011	-0.034
	P	0.313	0.590	0.529	0.844	0.505	0.716	0.285	0.905	0.722

Data are presented as correlation coefficient r and P value. vertical: vertical distance from the acupoint to the skin; +: vertical distance from the acupoint to the radius; needletipL, detailedL, needletipR, and detailedM: acupoint locations; CDFI: blood flow condition; MEAN: mean elasticity value; 1: acupoint site, 2: reference site.

Supplementary Figure

Supplementary Figure 1 On-site images from the multicenter technical operation training.

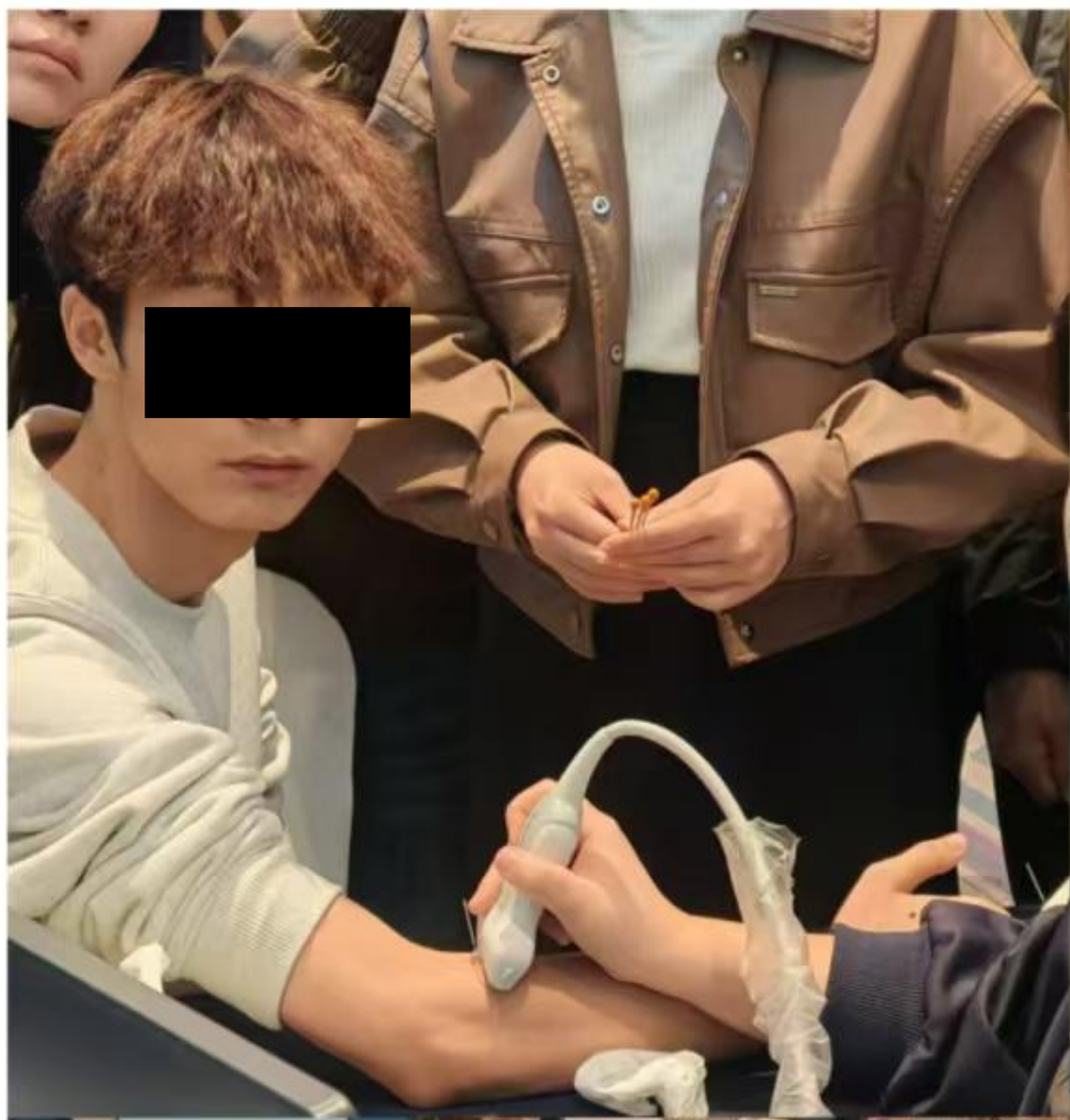
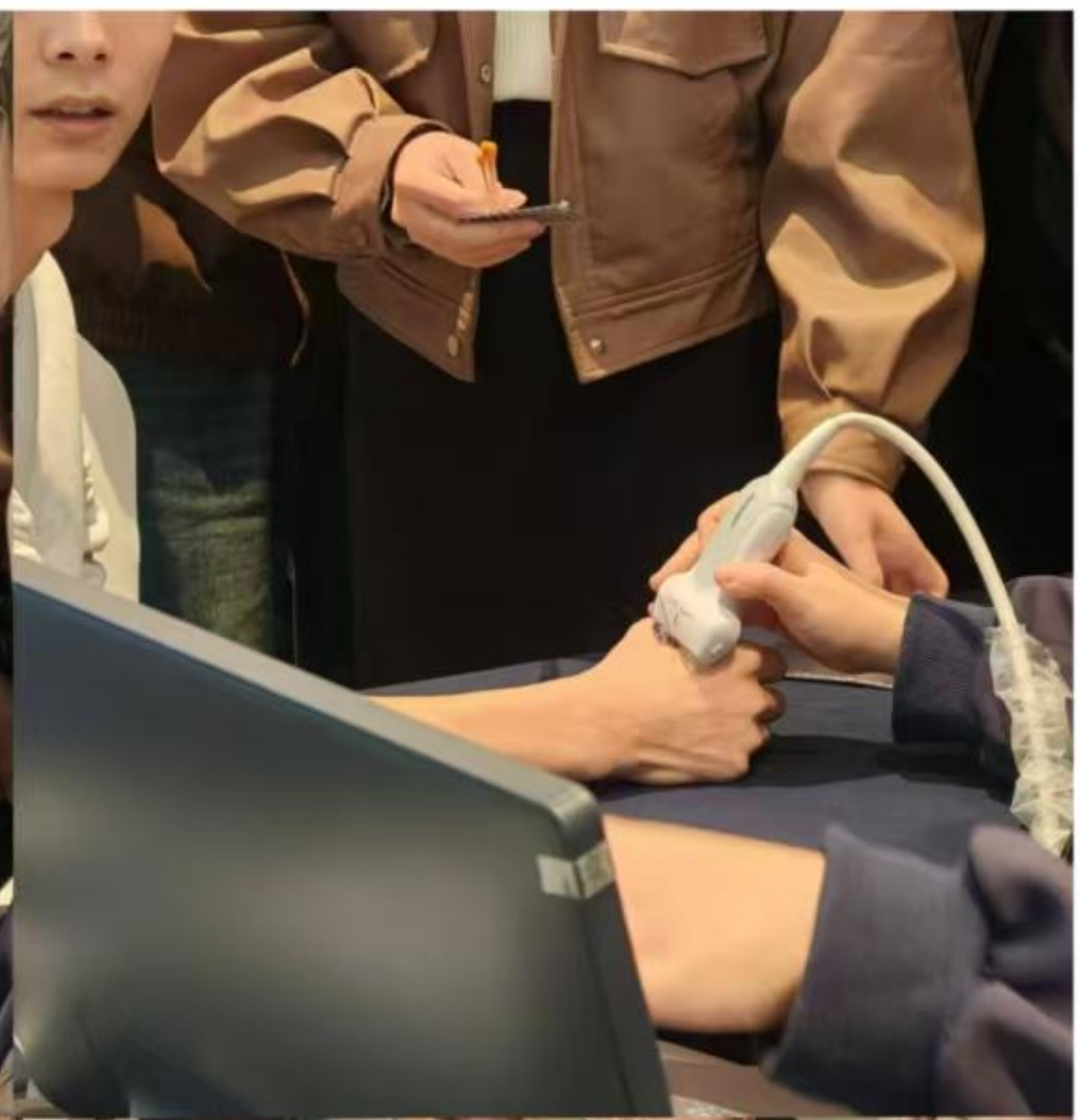
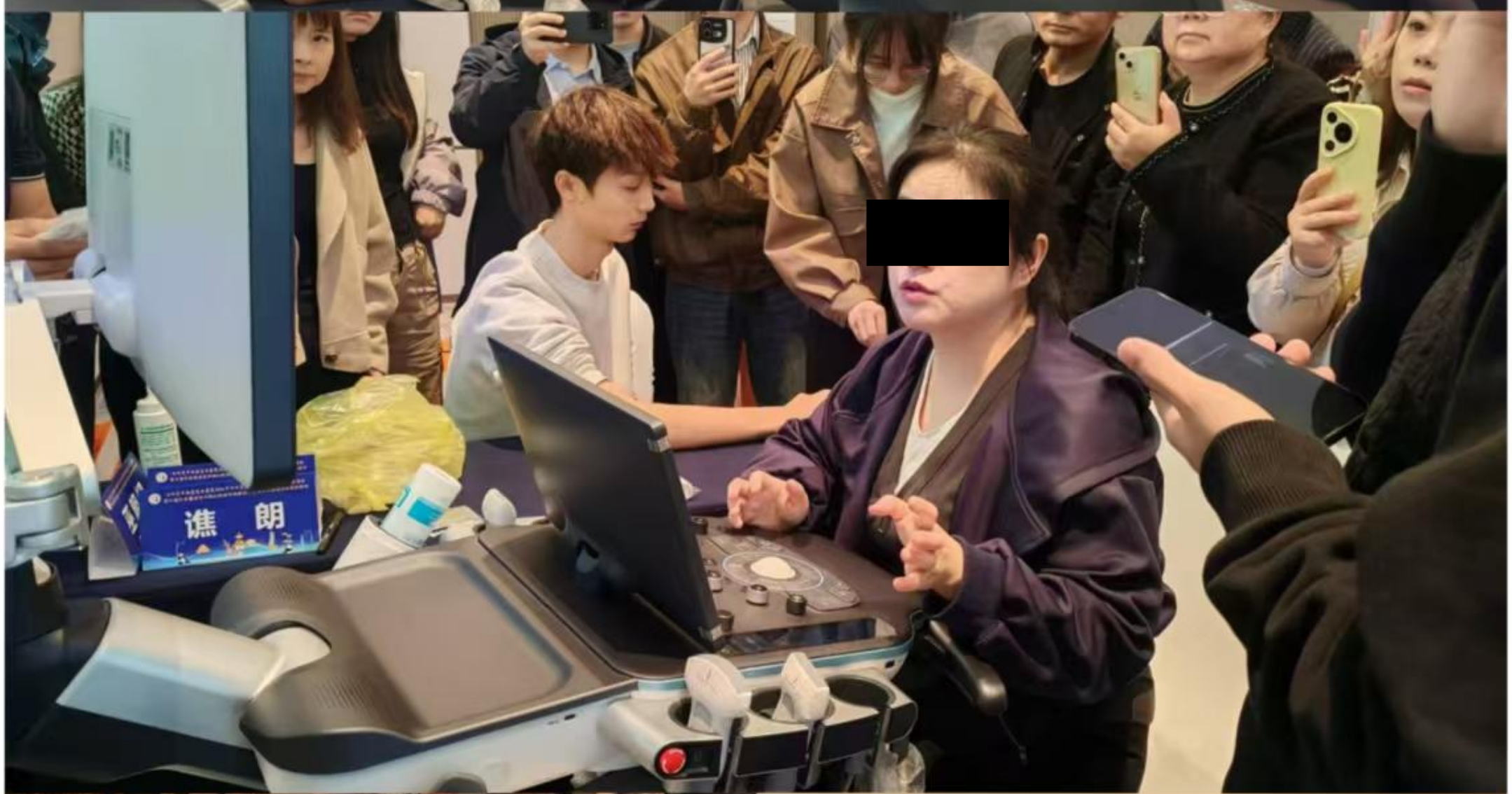
谯 朗
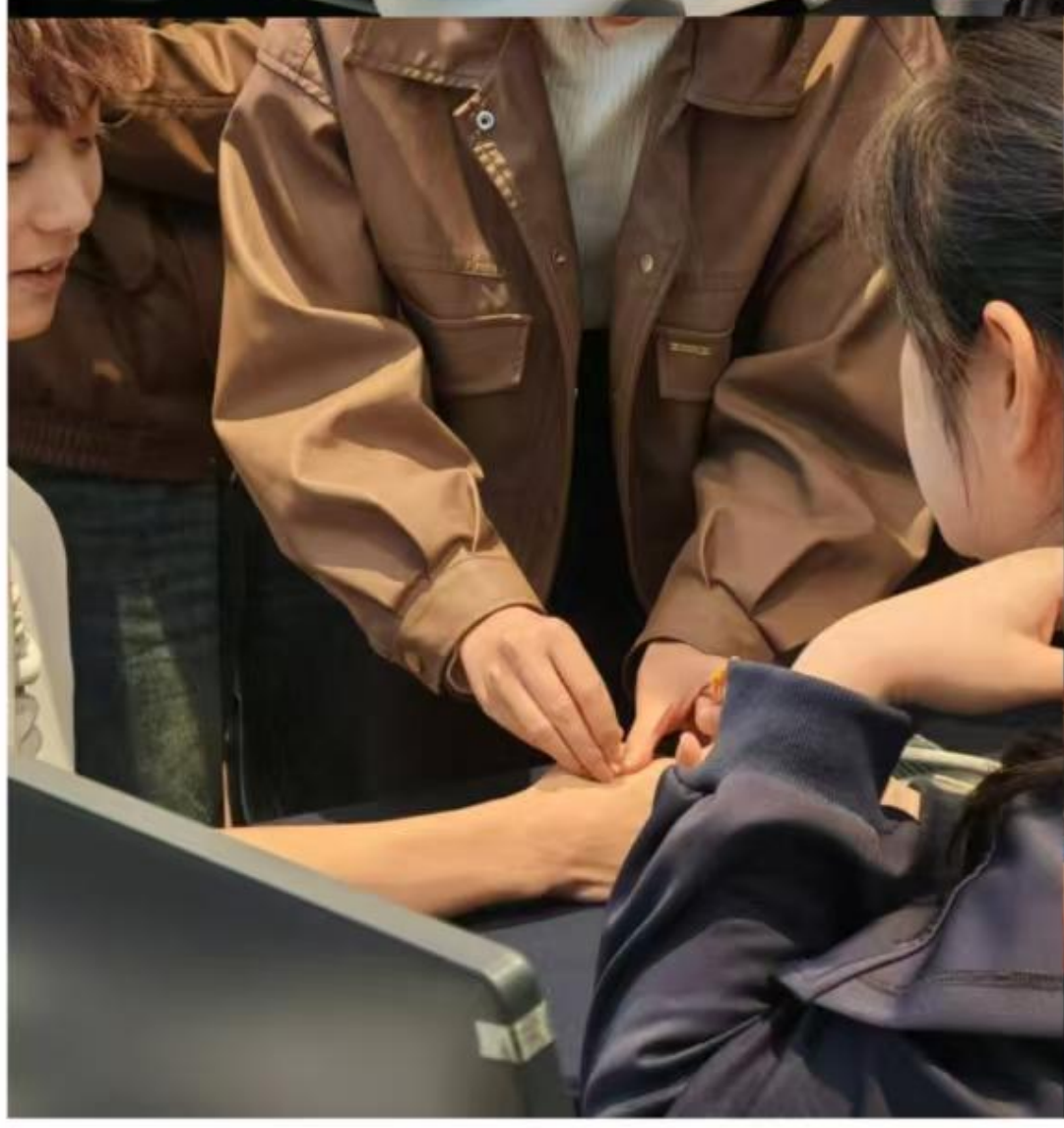
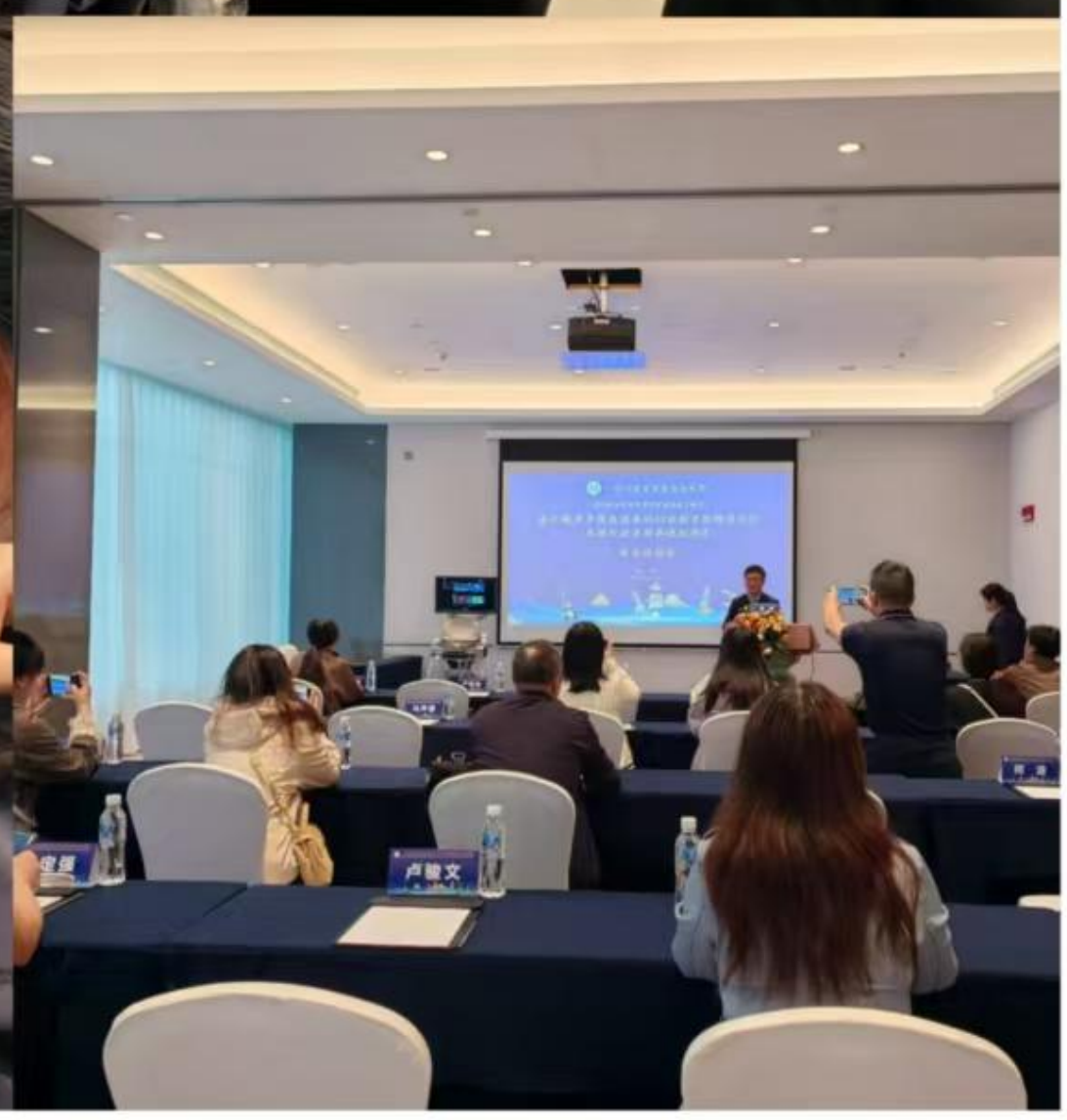
卢聪文

The operator in the middle picture (jiaojun yan) is the process of the PI Unit Training Sub-center
